# A Framework for Modeling Flood Depth Using a Hybrid of Hydraulics and Machine Learning

**DOI:** 10.1038/s41598-020-65232-5

**Published:** 2020-05-19

**Authors:** Hossein Hosseiny, Foad Nazari, Virginia Smith, C. Nataraj

**Affiliations:** 1 Department of Civil and Environmental Engineering, Villanova University, Villanova, PA 19085 USA; 2 Villanova Center for Analytics of Dynamic Systems (VCADS), Villanova University, Villanova, PA 19085 USA

**Keywords:** Hydrology, Natural hazards, Engineering

## Abstract

Solving river engineering problems typically requires river flow characterization, including the prediction of flow depth, flow velocity, and flood extent. Hydraulic models use governing equations of the flow in motion (conservation of mass and momentum principles) to predict the flow characteristics. However, solving such equations can be substantially expensive, depending upon their spatial extension. Moreover, modeling two- or three-dimensional river flows with high-resolution topographic data for large-scale regions (national or continental scale) is next to impossible. Such simulations are required for comprehensive river modeling, where a system of connected rivers is to be simulated simultaneously. Machine Learning (ML) approaches have shown promise for different water resources problems, and they have demonstrated an ability to learn from current data to predict new scenarios, which can enhance the understanding of the systems. The aim of this paper is to present an efficient flood simulation framework that can be applied to large-scale simulations. The framework outlines a novel, quick, efficient and versatile model to identify flooded areas and the flood depth, using a hybrid of hydraulic model and ML measures. To accomplish that, a two-dimensional hydraulic model (iRIC), calibrated by measured water surface elevation data, was used to train two ML models to predict river depth over the domain for an arbitrary discharge. The first ML model included a random forest (RF) classification model, which was used to identify wet or dry nodes over the domain. The second was a multilayer perceptron (MLP) model that was developed and trained by the iRIC simulation results, in order to estimate river depth in wet nodes. For the test data the overall accuracy of 98.5 percent was achieved for the RF classification. The regression coefficient for the MLP model for depth was 0.88. The framework outlined in this paper can be used to couple hydraulics and ML models to reduce the computation time, resources and expenses of large-scale, real-time simulations, specifically for two- or three-dimensional hydraulic modeling, where traditional hydraulic models are infeasible or prohibitively expensive.

## Introduction

Estimating spatial distribution of hydraulic characteristics of rivers for specific discharges, including water depth and water surface elevation (WSE)^1,2^, is critical in river engineering problems^3^. The quantification of such variables contributes to the solution of a variety of river management challenges, including river rehabilitation, recreation, sustainable ecosystems, and most importantly flood mitigation. Floods are one of the most devastating natural disasters. Due to climate change^4–6^ it is expected that floods will be more frequent and larger globally^7–10^, which means that river management will become increasingly challenging. An important step towards resilient flood control is to develop models that can identify areas vulnerable to flooding^11^. For flood mitigation, it is vitally important for the river manager to obtain a prediction of water depth and WSE accurately and promptly^12^. Prediction of any variable by models can be formidable due to computational expenses, a variety of uncertainties^5^ stemming from incomplete or noisy input data, measurement errors, inaccurate model calibration, and model simplifications^13^. This necessitates a need for robust models that can adapt efficiently to new scenarios with a range of variations in input data, and which can provide the best representation given the various uncertainties.

River hydraulic models are advanced physics-based models, capable of flow characterization in one, two or three-dimensional domains. The majority of such models solve a simplified version of the Navier-Stokes equation (NS), coupled with the conservation of mass equation, to formulate the motion of fluid^14^. NS is a mathematical formulation of the conservation of linear momentum in differential form through time and space^15^. Solving differential equations of conservation of mass and momentum for a large domain (national or continental scale) is tedious, time-consuming and costly. This makes the use of hydraulic models for large-scale simulations, specifically for fine-resolution topographic data, impractical. Currently, due to costs associated with hydraulic modeling, only 40% of the coterminous United States has been mapped by the U.S. Federal Emergency Management Agency (FEMA)^16^. Large-scale river modeling is extremely costly, mainly due to its computational expenses, which requires substantial simplifications in the modeling, and this demonstrates the need for more efficient modeling methods.

Likewise, real-time prediction of river hydraulics also demands more efficient modeling. At the moment, the National Water Model (NWM), a large-scale hydrologic-hydraulic model, predicts the weather and simulates the real-time flow and flood extent for 2.7 million river reaches in the continental United States^17^. The NWM estimates the effects that forecasted weather will have on discharges in streams and runs a simplified hydraulic model to predict real-time water surface elevation (WSE) in a continental domain. Such simplifications include the geometry of the streams and the hydraulics of the flow, by assuming a trapezoidal cross section for the streams, a quasi-normal flow condition, and a reduction in the flow computation dimension^18^. Such simplifications increase uncertainty and inaccuracy in predicted results^5^. Further, most of the hydraulic models are quite sophisticated and running them for new scenarios usually requires prior knowledge of the models and experience with them, which has been found to be highly challenging^19^. This makes the use of the hydraulic model for real-time simulations nearly infeasible, specifically for emergency responses^18^. Consequently, there is a great need for quick, robust, and versatile models for large-scale, real-time flood modeling.

In parallel with physics-based models, machine learning (ML) techniques have evolved through time^20^, focusing on learning from current data and experiences to enhance understanding of real-world problems^21^. Such approaches are specifically useful when (a) the current models are not fully capable of capturing the physics in mathematical terms, (b) the computational cost is impractical, or (c) the available knowledge about the problems is limited. This has made ML approaches powerful tools for assessing different aspects of water resources engineering, including, but not limited to, water distribution networks, water quality analysis, stage-discharge relations, sediment transport, rainfall-runoff estimation, flood susceptibility mapping, and flood prediction^20,22–32^.

Regression and classification are essential tools required for applications of ML in river engineering. While an ML classifier can distinguish between flooded and not flooded areas (wet and dry) over the domain with flow fluctuations, a regressor function can be used to estimate the depth of the flow in wet areas. The random forest (RF) model developed by Breiman (2001) is a powerful ML tool for classification problems. RF is a fast, efficient, and stable method for handling large data, even in the presence of missing data, as well as multicollinearity^31,33^. In addition, an important advantage of RF is that overfitting is not a major problem^33^. The RF is based on the decision tree algorithm and is composed of a combination of trees, in which each tree depends upon a randomly sampled vector of the dataset. The RF algorithm includes the selection of a random subset of the data for each tree and then the development of that tree at the node based on the best split of the data^16,33^. The RF algorithm includes the following steps: (a) training data is sampled several times, (b) random features are selected from each sampled dataset, (c) a decision tree is made based on the features of each sampled dataset, and (d) integration of the result of all the trees^34^. The classification decision is based on the majority of the votes estimated by all the trees. The RF has been successfully used for multiple flood-related problems, including mapping flood and landslide susceptibility^31,34,35^, finding the flood extent for gaps in flood inundation maps^16^, predicting flood damage^36^, and determining areas susceptible to flooding^37^. However, the application of RF for estimation of the flood extent, based on hydraulic analysis, has not been studied.

Among ML methods, the artificial neural network (ANN) model with the multilayer perceptron (MLP) tool is the most widely used approach^20,22,38–40^. MLP relates the input data to the output (target) through a network of neurons (calculation units) and shows a high level of connectivity between input, output, and neurons, enabling the model to capture the nonlinear behavior of the system^41^. This makes the MLP a powerful tool for modeling nonlinear stochastic systems, such as rainfall-runoff simulations, sediment transport quantification, and flood predictions^25–27,42–48^.

Physics-based models are inadequate for applications that require the results in a short time or for repetitive simulations^49,50^, specifically for large-scale modeling, due to their computational expenses. Such models are limited in evaluating uncertainty propagation in repeated simulations, and in forecasting real-time flood depth^50^. In these cases, well-trained ML models can replicate the results of physics-based models more efficiently^50,51^. Integrating physics-based models with ML methods has the potential to improve the quality of predictions^19^.

Currently physics-based, two-dimensional flood depth simulations are prohibitively expensive, specifically for large-scale simulations with high-resolution topographic data. Furthermore, the application of ML in flood modeling is in its early stages and needs substantial improvement^19^. To fill this gap, this paper outlines a novel hybrid of a river hydraulic model and selected ML techniques to estimate flood extent, river depth, and water surface elevation. The developed methodology specifically (a) uses an RF classifier to identify wet and dry nodes in the domain for the river discharge, and (b) uses an MLP model to estimate water depth in wet areas. The water surface elevation and flood extent over the domain for the wet nodes can be estimated based on these two steps. The proposed framework is a novel approach in flood characterization that will enable efficient, inexpensive, large-scale flood simulations. The outlined framework can be coupled with weather forecast models in the upstream which makes analyzing different *real-time* flood scenarios for two-dimensional large-scale simulations feasible. The outcome of this research is a quick, and flexible ML model that can efficiently identify flood characteristics including flooded areas, flood depth, and water surface elevation for different scenarios.

## Materials and Methods

### Study area

**Figure 1:**
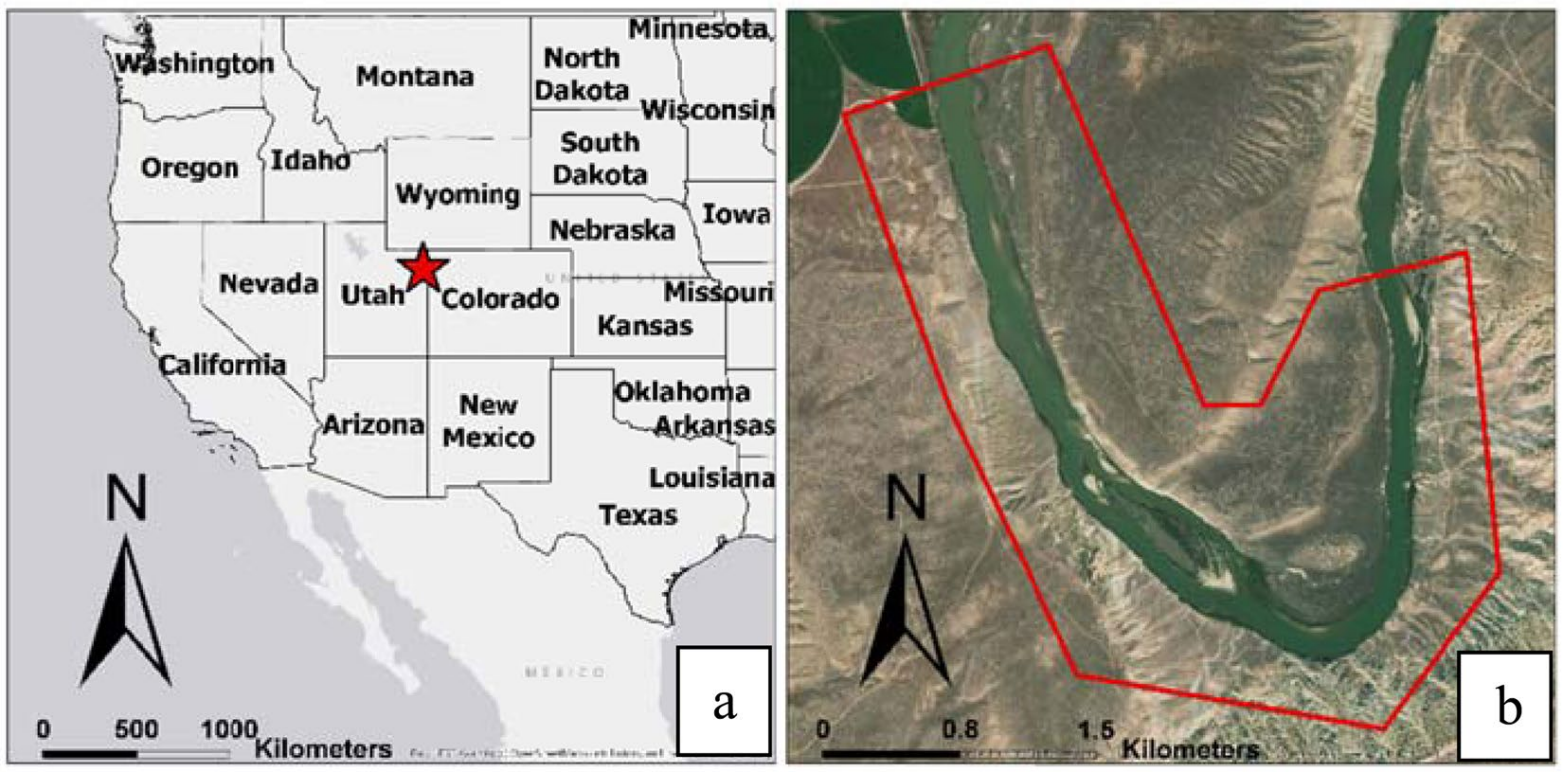
Global (**a**) and local (**b**) maps for the study area. The segment of the Green River selected for this study is shown within a red polygon. Maps were created in ESRI ArcGIS Pro version 2.4.0 (https://www.esri.com/en-us/arcgis/products/arcgis-pro/overview).

The study area used for this investigation is a 3.5 kilometers (km) segment of the Green River located at 120 km downstream of the Flaming Gorge Dam in the northeast corner of Utah, with the river width varying from 100 to 150 m (Fig. 1). The riverbed in this area is made up of a variety of bedrock lithology, forming fixed or restricted meanders^52^. The selected segment of the river for this study is sinuous, with a sinuosity coefficient of two, with random point bars (sediment deposition), which causes significant variations in water depth across and along the river in various discharges. For this segment of the river, bathymetry, and measured water surface elevation data have been collected by the United States Geological Survey (USGS), and are documented and available online to the public^53^. This makes it a good source for this study.

### Hydraulic model

**Figure 2:**
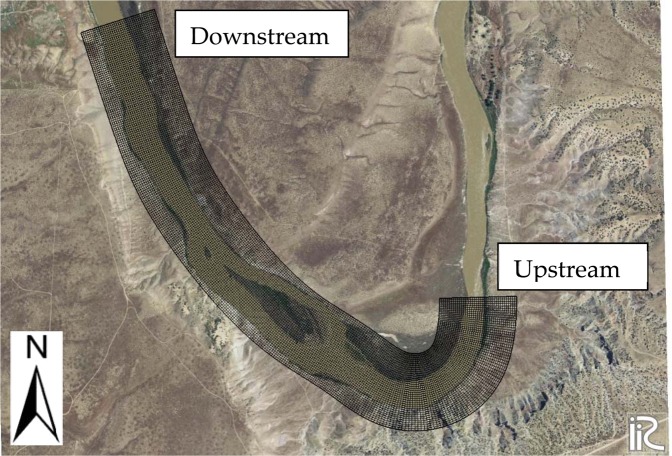
Meshed geographic data shows the computational domain in the iRIC. The grid cells are 5 (x) × 5 (y) meters. Figure was created in iRIC version 3.0.18, revision 6257 (https://i-ric.org/en/).

The International River Interface Cooperative (iRIC) is a two-dimensional hydraulic model with an integrated simulation solver. The input data to the model includes geographic data and measured hydraulic data (mainly water surface elevation) for model calibration. The iRIC model provides the user with the choice of solver selection. Different solvers in the iRIC simulate different hydraulic engineering problems, including, but not limited to, rainfall-runoff, river flow, sediment transport, morphological alterations, mudflow, and flow through culverts^53^. The solver selected for this study was FaSTMECH (Flow and Sediment Transport with Morphological Evolution of Channel). The geographic data input to the model included the coordinates and elevation of the points (*x*, *y*, *z*). Once the data was imported into the model, the mesh was generated and mapped for grids of 5 (*x*) × 5 (*y*) meters (Fig. 2).

### Model solver

FaSTMECH is a two-dimensional hydraulic model capable of addressing sediment transport in the domain. The model is quasi-steady, which implies that it can handle variations in discharge, while the unsteady terms in governing equations are neglected^54^.

### Mathematical background

The fundamental governing equations of fluid motion within the FaSTMECH include conservation of mass (Eq. 1) and conservation of momentum (Eq. 2) for an incompressible flow as follows:

1 $$\nabla .\,\overrightarrow{U}=0$$

2 $$\frac{\partial \overrightarrow{U}}{\partial t}+\overrightarrow{U}.\,\nabla \,\overrightarrow{U}=-\frac{1}{\rho }\nabla P+\overrightarrow{g}+\nu \,{\nabla }^{2}\,\overrightarrow{U}$$

in which $\overrightarrow{U}$ is the velocity vector, $\rho$ is the fluid density, *P* is the pressure, and ν is the kinematic viscosity of the fluid^14,15,54^. The solution of the above equations is usually carried out through some assumptions in order to simplify the equations. Such simplifications, which might include time averaging or averaging over one dimension, would be helpful in modeling turbulence flows, where a high degree of fluctuations occurs in both spatial and temporal domains. FaSTMECH uses Reynolds’ decomposition approach with respect to time (t), assuming

3 $$u(t)=\bar{u}+u{\prime} (t)$$

where $u\,(t)\,$ is the velocity, $\bar{u}$ is the time-averaged velocity, and ${u}^{\text{'}}$ is the velocity fluctuation. Equation 4 shows the Reynolds’ momentum formula for x direction as

4 $$\frac{\partial \bar{u}}{\partial t}+\bar{u}\,\frac{\partial \bar{u}}{\partial x}+\bar{v}\,\frac{\partial \bar{u}}{\partial y}+\bar{w}\,\frac{\partial \bar{u}}{\partial z}=-\frac{1}{\rho }\,\frac{\partial P}{\partial x}+\nu \,{\nabla }^{2}\bar{u}-\left(\frac{\partial \overline{{u{\prime} }^{2}}}{\partial x}+\frac{\partial \overline{u{\prime} v{\prime} }}{\partial y}+\frac{\partial \overline{u{\prime} w{\prime} }}{\partial z}\right)$$

where ρ and *P* are the density and pressure respectively, *u*, *v*, and *w* are velocity vectors, and *u*′, *v*′, and *w*′ are velocity deviations from time-average velocity in *x*, *y* and *z* directions. The pressure term (*P*) in Eq. 4 is assumed to be hydrostatic, implying that the vertical pressure distribution is only related to the weight of the column of water^54^. FaSTMECH solves the governing equation of fluid motion for a wide variety of coordinate systems, including cartesian and orthogonal curvilinear systems with structured and unstructured meshes. The orthogonal curvilinear coordinate system allows the curvature of the coordinate system to vary in stream-wise direction, providing a channel-fitted coordinate system. The unstructured mesh enables the model to fit precisely to the flow domain but increases the computation complexity significantly^53^.

### Model calibration

**Figure 3:**
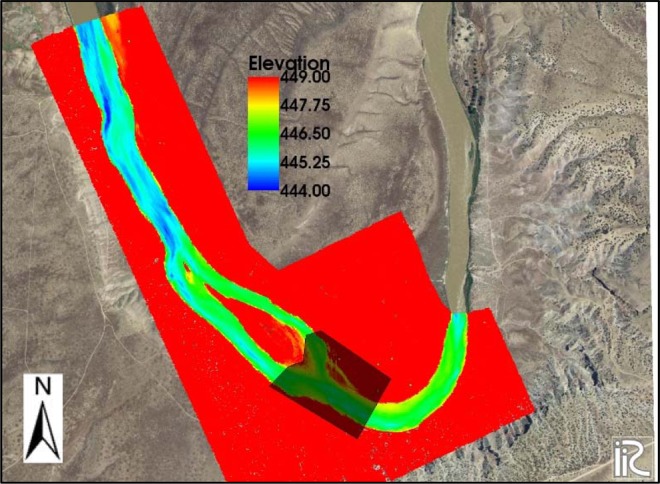
An increase in roughness before the sediment island (area within the darker polygon) increased the accuracy of the FaSTMECH model in predicting the measured water surface profile. Figure was created in iRIC version 3.0.18, revision 6257 (https://i-ric.org/en/).

The calibration of hydraulic models usually includes setting up the input variables (mainly the roughness coefficient) so that the model can accurately generate a measured water surface profile for a known discharge. For the study area, the model calibration has been documented by the USGS, which included the data and the guidelines provided within the iRIC manual and the FaSTMECH Tutorial 2^53^. The initial conditions for the model simulations included the known discharge of 247 cubic meters per second (m^3^/s) upstream and the known water surface elevation of 447.1 meters downstream. The roughness coefficient in the iRIC is considered in the form of the drag coefficient. Initially, a sensitivity analysis was done to estimate the root mean square error (RMSE) of the predicted and measured water surface profile by a change in the drag coefficient, which varied from 0.005 to 0.1. The drag coefficient of 0.008 resulted in minimum RMSE, and therefore it was selected for the whole domain. However, a further adjustment was recommended by the USGS to decrease the error even more. The adjustment included an increase in the drag coefficient of the riverbed before the sediment bar (island) from 0.008 to 0.013 which decreased the RMSE from 0.05 to 0.03. This implies that flow separation around the sediment bar increased the roughness due to flow separation and energy loss. The red polygon in Fig. 3 shows the domain of topographic data for the study area with the drag coefficient of 0.008, and the darker polygon depicts the area with increased roughness to 0.013.

**Figure 4:**
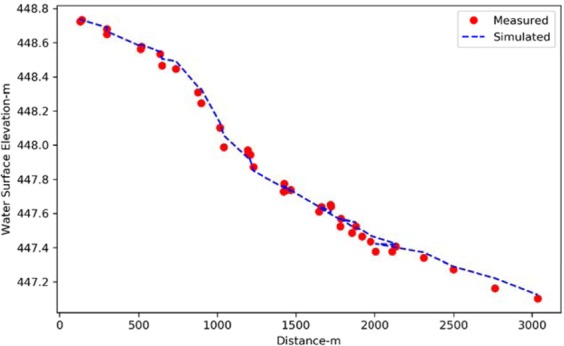
Comparison between measured and simulated water surface elevation along the river in the study area for a discharge of 247 m^3^/s.

Figure 4 shows simulated water surface profile along with measured WSE along the segment for a discharge of 247 m^3^/s, which illustrates a well-calibrated model.

### Hydraulic simulation

The historic peak discharges of the river from 1951 to 2018 were obtained from the USGS gage located downstream of the Flaming Gorge Dam (USGS 09234500 Green River near Greendale, UT). The historic peak flows varied from 20 to 560 m^3^/s. For the purpose of training the ANN model, a broadly representative set of discharges were selected for simulation. Seven river discharges of 10, 50, 95, 120,150, 300 and 400 m^3^/s were chosen to represent low, medium and high flows. The simulation results for each run included the mesh node coordinates, two-dimensional velocity and shear stress, water depth, ground elevation, and whether the node was wet or dry (0 or 1).

### Machine learning

**Figure 5:**
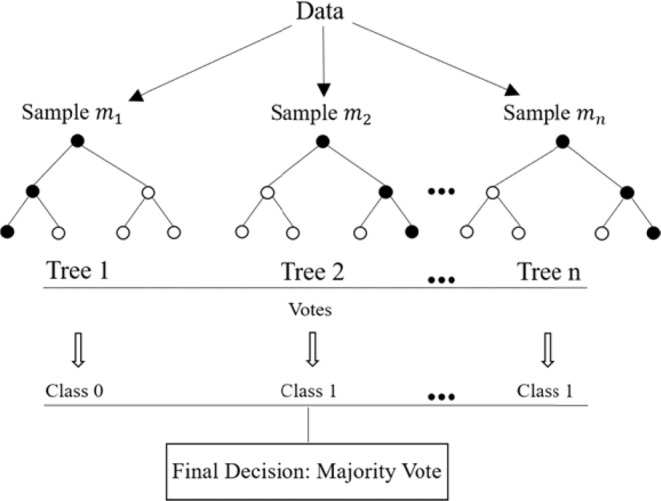
Schematic of the RF and how the final decision is made by the majority of the votes.

The quantification of the flood extent and depth by the ML framework was tackled in two steps. First, the RF model was used to identify wet areas in the domain for a specific discharge. Secondly, the ANN model was used to estimate the depth in wet areas. The RF is constructed by a combination of decision trees, in which each tree acts on a randomly selected vector of the dataset (sample). The model contains several trees. Each tree is a calculation unit in which the input data is processed, and the decision is generated at each tree. The final decision on the classification type is made by the majority of the votes (Fig. 5). In that sense, trees in the RF are similar to the neurons in the MLP.

An increase in the number of trees normally increases the accuracy of the model^33^. For each increase the RF produces an unbiased error generalization that prevents it from overfitting^33,55^. In the RF algorithm, two random selections of the data take place in the training process. First, two-thirds of the data are randomly selected with replacement. The remaining one-third of the data (out-of-bag) is used to monitor the error in training. This step provides the RF with a parallel cross-validation which ensures a higher accuracy^56^. The second random selection takes place in the tree nodes, where a subset of the input variables is selected. This maximizes the split of the data^16^. More information about RF can be found in the publication by Breiman (2001), where the technique was initially introduced.

**Figure 6:**
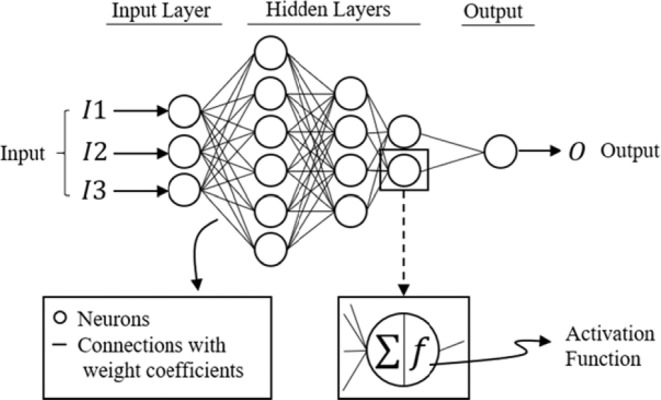
Schematic of an ANN model and the connection between the neurons.

Artificial neural network (ANN) is a data processing paradigm that mimics the biological structure of the human brain and is used to learn and capture the behavior of highly complex systems. The multilayer perceptron (MLP) is one of the most widely used ANN architectures for both regression and classification. MLP is composed of the input and output layers with some arbitrary hidden layers between them. Each hidden layer contains several neurons (Fig. 6).

The neurons are the calculation units that receive the input data, process them, and generate the output using the activation function. While weight connections are all linear, the activation function transforms the output to a nonlinear form, which enables the ANN to capture the nonlinearity of problems. The capability of the ANN to understand the behavior of a system is embedded in the weights of the connections between neurons. The ANN is trained by tuning the connection weights so that the model can mimic the behavior of the actual system. There are various methods for training the ANN, including back error propagation (BEP), the most extensively used supervised training algorithm. The BEP method includes feed-forward, back error propagation and weight adjustment stages^41^. For this study, the gradient descent training function was used. All data were preprocessed by standardization (scaling the data from zero to one) before introducing them to the neural network.

The activation function used in this study was set to the hyperbolic tangent-sigmoid (Tansig) transfer function which is defined as

5 $$T(\tilde{x})=\frac{2}{1+{e}^{-2\tilde{x}}}-1$$

where $\tilde{x}$ is the weighted sum of the inputs to the neuron, and $T$ is the output from a neuron. Error function (loss function) measures the error between output and target, which is used in training processes to adjust the ANN weights. In this study, the mean square error (MSE)^57^ function is used to estimate the error values for classification and regression.

### Hybrid hydraulic-ML approach

**Figure 7:**
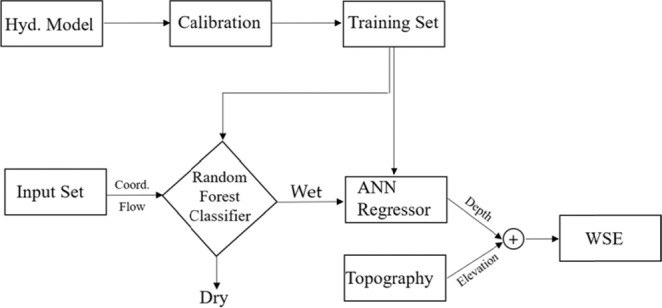
The flow chart of the hybridization of hydraulics and ML for predicting river depth and WSE.

This section outlines the methodology and suggested a framework for river modeling. The framework demonstrates how hydraulic and data-driven models can be used to (a) identify potentially flooded areas and (b) to estimate the probable depth of flooding in such areas. A general overview of the proposed approach is summarized in Fig. 7, where WSE is the water surface elevation relative to a datum.

Figure 7 shows the steps for modeling the river flow with ML tools based on the training dataset generated by the hydraulic model. The hydraulic model is calibrated based on measured water surface elevation.

To identify the river depth by the ML approach the following steps were developed:

- RF was used to identify wet (W) and dry (D) nodes (1 and 0 respectively) in the domain based on the input discharge and the location coordinates in the domain.
- ANN was used to develop a model to estimate the depth of the flow over the domain for an arbitrary discharge and the location in the domain (coordinate *x, y*). The estimated depth can then be used along with topographic data to estimate the water surface elevation.

Once the depth is estimated, the water surface elevation for wet areas can be estimated by adding up the known ground elevation data of each location to its estimated water depth.

## Results and Discussion

### Hydraulic analysis

River discharge is one of the most important factors that determine river depth. Depending upon the geometry of the river and the resistance against the flow, the depth can vary considerably with a change in river flow. This section aims to show the depth variations in the study area due to changes in river discharge. To do that, the relative magnitude of the low, medium, and high discharges were identified based on the percentile of exceedance of the historic mean daily discharges^58,59^. Mean daily discharge data for the recent two decades (from January 1, 1999 to December 31, 2019) were obtained from the USGS gage 09234500 of the Green River near Greendale and were used to estimate the exceedance discharges for 2, 50, 84, and 98 percentiles^60^. Table 1 shows the exceedance percentiles and their associated discharges, indicating the low to high discharges.

**Table 1:** Percentile exceedance of the discharge data.

Percentile Exceedance	2%	50%	84%	98%
Discharge (Q)-m^3^/s	23	39	64	238

Based on the estimated exceedance values, five sets of discharge data were selected to represent extremely low to extremely high discharges, as tabulated in Table 2.

**Table 2:** Selected discharge data for the test (Q_test_) based on the discharge statistics.

Relative Flow Magnitude	Flow Exceedance condition	Q_test_ (m^3^/s)
Extremely High	Q_exceeded_ < 2%	350
High	2% < Q_exceeded_ < 16%	225
Moderate	16%< Q_exceeded_ < 50%	45
Low	50%< Q_exceeded_ < 98%	30
Extremely Low	Q_exceeded_ < 98%	20

**Figure 8:**
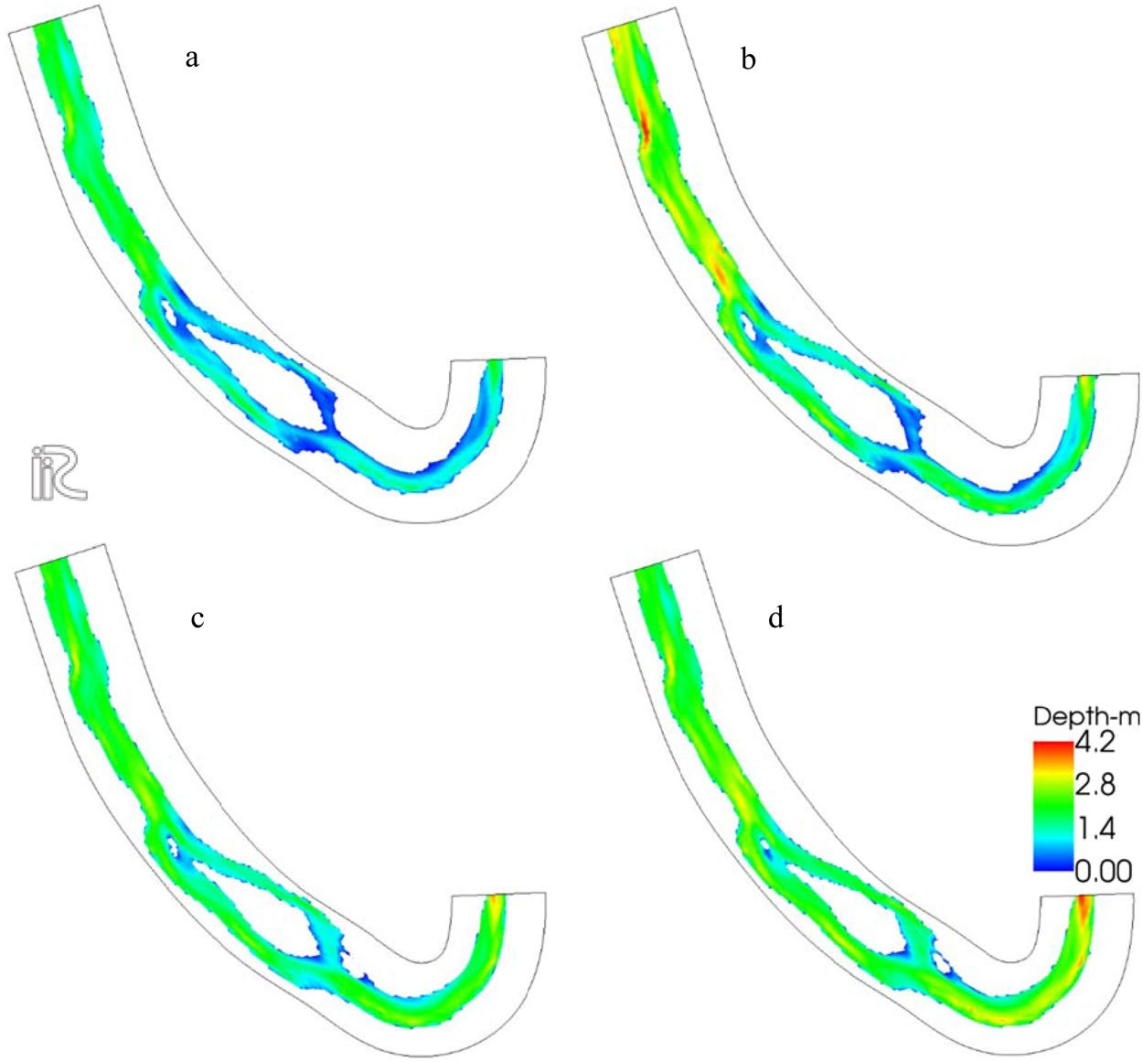
FaSTMECH simulation results show the flood extent and the depth for discharges of (**a**) 30 (b) 45 (**c**) 225, and (**d**) 350 m^3^/s. Figures were created in iRIC version 3.0.18, revision 6257 (https://i-ric.org/en/).

The calibrated FaSTMECH model was run for discharges of 20, 30, 45, 225 and 350 m^3^/s. The extreme peak discharge of 350 m^3^/s was chosen to magnify the visualization of the spill of the flow over the bank (bank activation) during large floods. The computation time for each discharge was 5 minutes. The simulation results, including the flood extent and depth associated with low, moderate, high, and extremely high discharges (30, 45, 225, and 350 m^3^/s), are depicted in Fig. 8.

**Figure 9:**
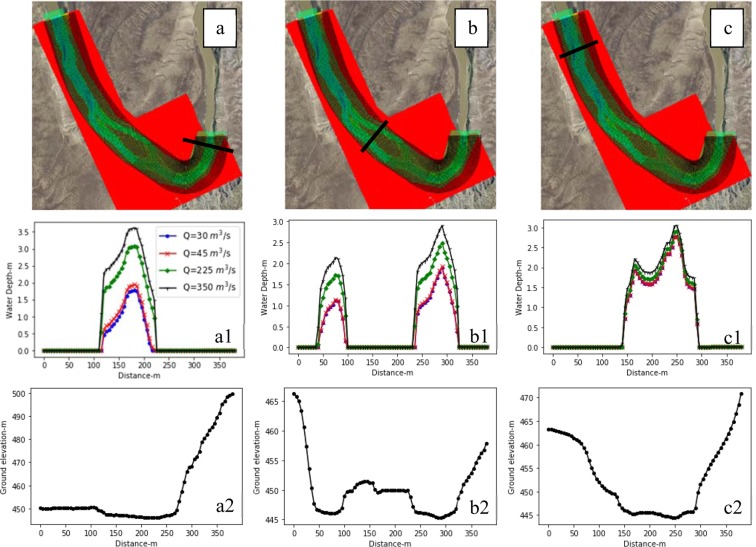
Three cross sections in (**a**) the upstream located at 0.1 river kilometers (river km), (**b**) midstream over the sediment bar at 1.25 river km, and (**c**) downstream at 2.45 river km, along with the variations in depth across the cross sections for river discharges of 30, 45, 225, and 350 m^3^/s are depicted. Maps a, b, and c were created in iRIC version 3.0.18, revision 6257 (https://i-ric.org/en/).

The model results showed that in general, depth increases with an increase in discharge. In the upstream cross section, the increase in discharge to 350 m^3^/s caused the water depth to rise to 3.5 meters (Fig. 9a). At the mid-stream cross section, the sediment bar did not get flooded at a high flow discharge of 350 m^3^/s. To better show the depth variations due to a change in discharge over the domain, three cross sections in upstream, mid-stream on the sediment bar, and downstream of the study area were defined (Fig. 9a–c). The bed elevation, along with the depth values associated with each discharge over three cross sections, were obtained and are depicted in Fig. 9. The flow depth around the sediment bar varied significantly. For instance, the maximum depth associated with the maximum discharge at the right branch of the river was 2.8 meters as opposed to 2.2 meters in the left branch (Fig. 9b). The maximum depth occurred in the commencement of the study area, which might be due to the effects of the inlet boundary conditions. The rate of the change in flow depth decreased downstream due to the change in discharge, which implied a smooth flow routing along the river.

The results showed that the river depth in the upstream cross section was more sensitive to the change in discharge than the lower parts. More specifically, the depth in the cross section downstream did not significantly change with the variation of discharge from 30 to 350 m^3^/s (Fig. 9 c1). Moreover, the river depths associated with the discharges of 30 and 45 m^3^/s were closer to each other than the others.

### Hybrid approach

#### Wet-Dry classification

**Figure 10:**
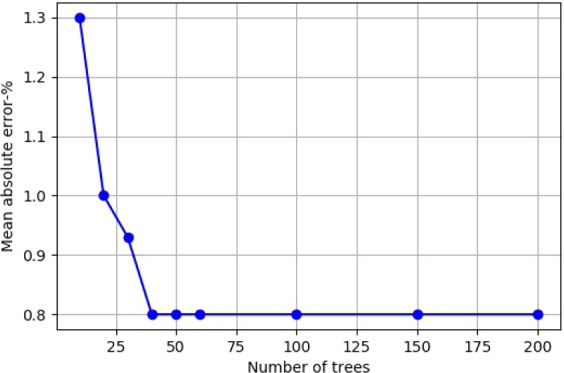
The relation between the number of trees and the mean absolute error for wet-dry classification.

Training the RF was carried out based on the wet and dry classification by the FaSTMECH for the discharges of 10, 50, 95, 120, 150, 300, and 400 m^3^/s. The wet-dry classification complexity was adequately reflected in the RF model with 50 trees. Increasing the number of trees above 50 did not increase the accuracy. Figure 10 shows the variations in mean absolute error generated by changes in tree numbers.

Seventy percent of the data was used for model training and the rest of the data was used for the test. The test resulted in an accuracy of 98.5%.

The performance of a binary classifier can be measured by multiple conventional statistical indices such as F1-score, Mean Absolute Error (MAE), and Root Mean Square Error (RMSE). Moreover, True Positive (TP), False Negative (FN), True Negative (TN), and False Positive (FP) indices show the performance of the model in quantitative terms^31^. TP is defined as the number of samples in the positive class which are correctly identified as positive, and FN is the number of samples that belong to the positive class and are incorrectly classified as negative. However, evaluating the performance of a classifier relying just on these indices might be misleading. For instance, a classifier might perform with a low misclassification rate in one class, and with a high rate in another class, which could pose a problem in some applications^61^. Thus, some other indices including sensitivity, precision, and overall accuracy can be used to evaluate the performance of a classifier. The sensitivity and precision metrics for class $i$ are defined as

6 $${S}_{i}=\frac{T{P}_{i}}{T{P}_{i}+F{N}_{i}}$$

7 $${P}_{i}=\frac{T{P}_{i}}{T{P}_{i}+F{P}_{i}}$$

The sensitivity shows the ability of the model to label all the positive samples correctly, and the precision shows the ability of the model not to label a negative sample as positive^62^. Furthermore, overall accuracy can be defined as

8 $$Overall\,Accuracy=\frac{T{P}_{all}}{n}=\frac{TP+TN}{n}\,$$

where, $\,T{P}_{all}$ indicates the total number of test points that have been classified correctly in their classes, and $n$ is the total number of test samples.

For any specific class, if the classifier presents high precision and low sensitivity, it means that the classifier is very conservative. On the contrary, high sensitivity and low precision for a given class show that the classifier is biased to that class. An ideal classifier is one that can present high precision and high sensitivity for all classes^61^. The F1-score, which is defined as the harmonic mean of precision and sensitivity, can be formulated as

9 $$F1\mbox{--}score=\left(\frac{2}{{{S}_{i}}^{-1}+{{P}_{i}}^{-1}}\right)=2\frac{{S}_{i}\times {P}_{i}}{{S}_{i}+{P}_{i}}$$

The closer the F1-score to a value of one, which is considered the maximum, demonstrates the best discrimination between two sets of samples^62,63^.

In classification problems, one of the most common ways to describe the performance of a classifier is the confusion matrix. The confusion matrix for the RF method used in this study is tabulated in Table 3. The numbers of the data which are classified correctly are shown on the main diagonal, and incorrectly classified data are shown in the off-diagonal arrays. For example, in Table 3, RF correctly predicted 60,426 points as wet and 36,951 points as dry. It also misclassified 1,422 points out of the 98,799 total data points. The RF method correctly classified 61.2% of test points as wet and 37.4% as dry. Thus, 0.7% of test points were incorrectly classified for each class. By comparison, the iRIC model classified 61.9% of the data for the wet points and 38.1% for the dry points.

**Table 3:** Confusion matrix for wet-dry classification of the training discharges.

		Confusion Matrix		Sensitivity	Precision
Output	Wet	60,426	709	98.8%	98.8%
	Dry	713	36,951	98.1%	98.1%
		**Wet**	**Dry**	**Overall Accuracy**	
		**Target**		**98.5%**	

For the binary classification of wet or dry in this study, wet conditions are considered as a positive class and dry as a negative class. Therefore, TP shows that the classifier correctly distinguishes that a test point is wet, and TN indicates the correct classification of the dry points. Also, FP and FN are indications of incorrectly placing a test point in wet and dry classes, respectively.

As shown in Table 3, the sensitivity and precision of the wet and dry conditions were 98.8% and 98.1% respectively. Furthermore, 98.5% of unseen test data was correctly classified (overall accuracy), which is a remarkable achievement. All these metrics show that the proposed approach has excellent predictability of whether any specific part of the river would get flooded for any given river flow. One of the potential applications of this classification model is to identify the areas which might be affected by expected flooding.

To evaluate the performance of the RF model in identifying wet nodes for another set of unseen data with finer mesh (1 (*x*) × 1 (*y*) meters), the RF model was run for the test discharges presented in Table 2. The test discharges were not used in the training process of ML models. Table 4 shows the accuracy of RF for identifying wet nodes for different discharges.

**Table 4:** RF classification accuracy for unseen discharges.

Q- m^3^/s	20	30	45	225	350
Accuracy-%	98.6	98.2	98.7	98.4	98.7
F1-score	0.96	0.96	0.97	0.98	0.99
MAE	0.05	0.05	0.04	0.02	0.01
RMSE	0.22	0.22	0.19	0.15	0.10

**Figure 11:**
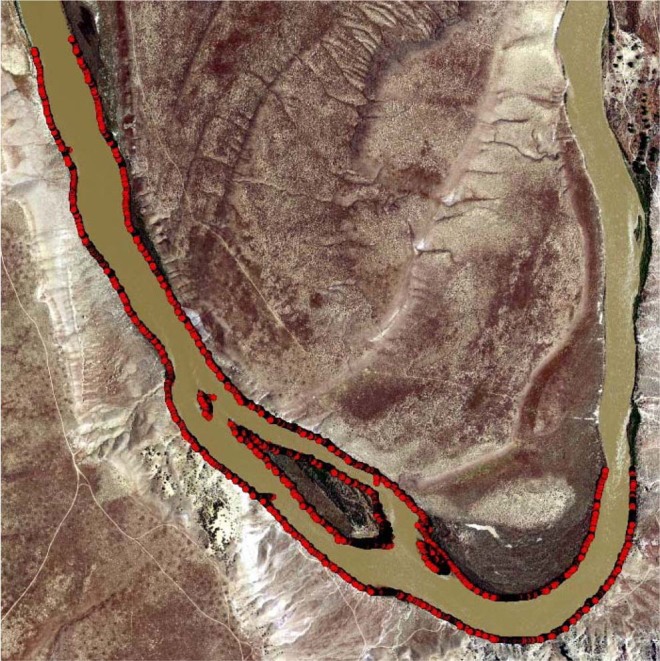
Red dots show the area where RF failed to classify wet and dry correctly for a discharge of 350 m^3^/s. These areas mainly consisted of the edges between land and water. Map was created in ESRI ArcGIS Pro version 2.4.0 (https://www.esri.com/en-us/arcgis/products/arcgis-pro/overview).

For the discharge of 350 m^3^/s and one-meter mesh size, the total number of input data was 1,113,371 points. The computation time for each discharge was 1.9 seconds. Figure 11 shows where the RF failed to classify wet and dry nodes correctly for the discharge of 350 m^3^/s, which mainly consisted of the edges between dry and wet areas.

#### River depth prediction

**Figure 12:**
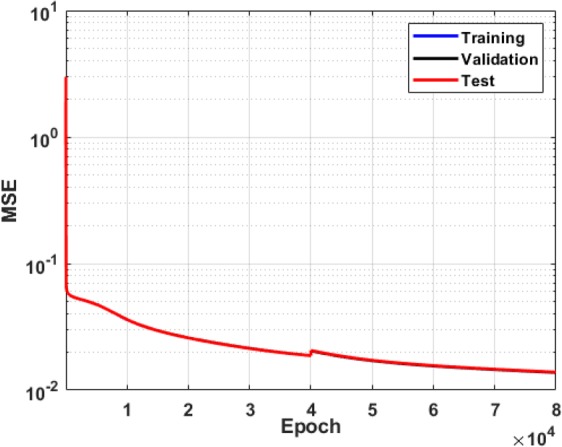
ANN training performance for depth prediction.

Due to the high complexity of the system, a single hidden layer neural network could not capture the inherent non-linearity of river depth variation. Therefore, the effects of the MLP hyper-parameters, including the number of hidden layers and neurons, on the prediction accuracy were investigated. As a result, the MLP model structure was set to four hidden layers with 150, 100, 50, and 30 neurons respectively. The error analysis showed that the complexity of the problem was adequately reflected in the proposed structure of the ANN model. The activation function for all layers was Tansig (Eq. 5). For training, the overall data number of the discharges of 10, 50, 95, 120, 150, 300 and 400 was 329,329, and the epochs numbered 80,000. Seventy percent of the data was used for training, twenty percent for validation, and ten percent for the test of the ANN. The error function was the mean square error. The training function for ANN was gradient descent with moment backpropagation. The regression coefficient is the slope of the linear regression line between the ANN model output and the target values which vary between zero and one. A regression coefficient closer to one indicates a more accurate training process. Despite the fact that the spatial variation in river depth is complex and highly nonlinear, the regression coefficient for this study was 0.88. The performance plot for this ANN is depicted in Fig. 12, which shows that the mean square errors for training, testing and validation were less than 0.02.

**Figure 13:**
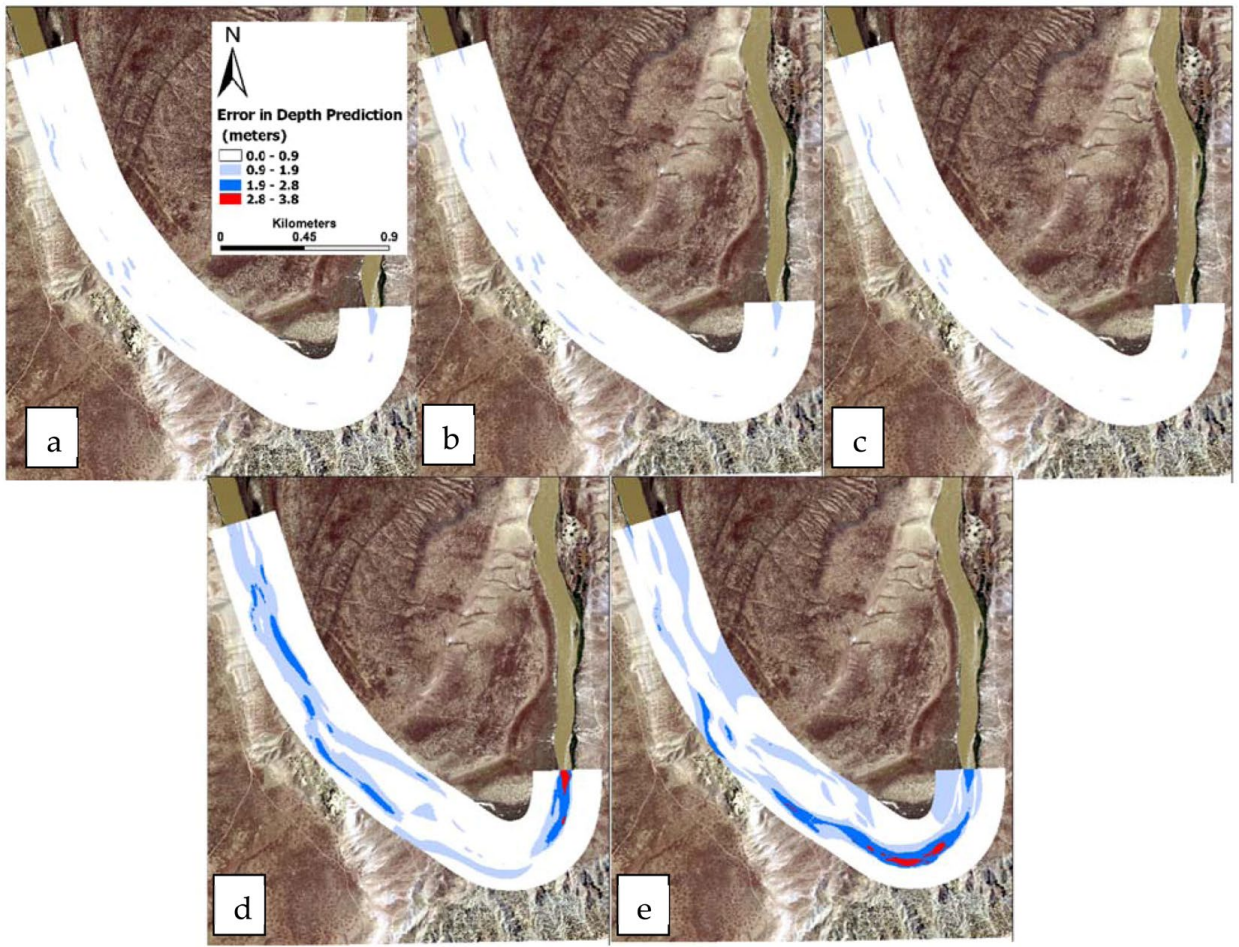
The difference between predicted depth by the ANN and the iRIC (defined as the error of predicted depth by ANN) is depicted for different flow discharges of (**a**) 20, (**b**) 30, (**c**) 45, (**d**) 225, and (**e**) 350 m^3^/s. Maps were created in ESRI ArcGIS Pro version 2.4.0 (https://www.esri.com/en-us/arcgis/products/arcgis-pro/overview).

After training, the ANN model was used to estimate the depth over the wet nodes for the discharges of 20, 30, 45, 225, and 350 m^3^/s. The ANN simulation time for each discharge was 5 seconds, as opposed to 5 minutes for a hydraulic simulation. The difference between predicted depth by the ANN model and simulated depth by the iRIC (defined as the error of predicted depth by ANN) for different discharges is depicted in Fig. 13. The lighter colors show smaller errors while dark blue and red show where the maximum error in depth prediction took place.

The analysis of the training, testing, and cross validation of the ML models used in this research showed that the overall accuracy of the classification model (Tables 3 and 4) and the mean square error for the river depth regression model (Fig. 12) were acceptable. The results showed that the error in depth prediction was highly variant with the change in river flow. The maximum error occurred at the highest discharge (Fig. 13e), and the minimum error was associated with the minimum discharge (Fig. 13a). It was also shown that the maximum error in depth prediction by the ANN model occurred in the river bend. For low and moderate flows up to 45 m^3^/s, the error in depth prediction was less than 0.9 meters all across the domain (Fig. 13a–c).

Obtaining an accurate WSE requires flow depth and data for topography (bed elevation). The WSE is the summation of the estimated river depth and known bed elevation for each data point. Therefore, the error in estimated WSE would essentially be the same as the error associated with predicted depth.

## Conclusion

Large floods are expected to occur more frequently around the globe due to global warming, which demands a new paradigm of robust, efficient, and real-time flood modeling. For most river engineering problems, including flood mitigation, there is a need for quantification of the river depth for different flow discharges. While numerous hydraulic models are capable of addressing these problems, large-scale hydraulic simulation, including two or three-dimensional flow with high-resolution topographic data, is computationally expensive in a way that discourages simulations. The aim of this paper is to propound a versatile framework for river flood modeling that can be used for large-scale two or three-dimensional flood simulations with high resolution topographic data. Currently, such simulations with traditional hydraulic models are practically unattainable.

This research outlines a novel framework that incorporates machine learning (ML) approaches for river depth prediction, using the Green River of Utah as a case study. The framework includes a random forest (RF) classification model that identifies wet and dry nodes, which can be used to identify affected areas by flood and WSE estimation. Moreover, the framework outlines an ANN model that predicts the river depth for any discharge over the study area. The ANN model incorporates the coordinates of any desired location over the domain, along with the discharge, and computes the estimated depth. The random forest classifier predicts where there would be flooding in the domain (wet nodes). The iRIC hydraulic model with the FaSTMECH solver (two-dimensional, quasi-steady hydraulic model) was used to generate data for training and evaluating the performance of both ML models.

The ANN model resulted in a regression coefficient of 0.88 with a mean square error of less than 0.02. The RF model resulted in an accuracy of more than 98 percent. Moreover, five sets of unseen data with the discharges of 30, 70, 135, 225, and 350 m^3^/s and the mesh size of one meter (more than 1.1 million data points) were used to evaluate the performance of the RF, which resulted in an overall accuracy of more than 98 percent in wet-dry classification. The computational time for RF classification was 1.9 seconds for each discharge. To evaluate the performance of the ANN model for depth prediction, the same five sets of data were run. The computational time for each discharge was 5 seconds. The results showed that proposed ML models can successfully predict flow depth as well as flood extent. Moreover, the ML approach reduced the computation time 60-fold. The computational time reduction is expected to be even more significant for large-scale simulations. The maximum error for depth prediction, which took place in the river bend, increased with an increase in river discharge.

Training the ANN model with finer mesh along with more training data is expected to increase the accuracy of the model for depth prediction, which can be pursued in the future, depending upon the availability of enhanced computational power. Moreover, to increase the accuracy of the results, the proposed framework can serve as a starting point for flood depth modeling with enhanced ML tools, such as deep learning and convolutional neural networks. The outlined framework makes feasible the analysis of real-time flood scenarios for large-scale simulations. Moreover, the framework can be coupled with weather forecast models in the upstream to estimate the river depth and flood extent downstream efficiently and effectively for real-time scenarios.
